# Complete Genome Sequence of *Effusibacillus* sp. Strain skT53, Isolated from Farm Soil

**DOI:** 10.1128/MRA.00481-21

**Published:** 2021-07-22

**Authors:** Tomoyuki Konishi, Tomohiko Tamura, Toru Tobita, Saori Sakai, Namio Matsuda, Hisashi Kawasaki

**Affiliations:** a Graduate School of Engineering, Tokyo Denki University, Tokyo, Japan; b National Institute of Technology and Evaluation, Kisarazu, Chiba, Japan; c Agro-Biotechnology Research Center, Graduate School of Agriculture and Life Sciences, The University of Tokyo, Tokyo, Japan; d Collaborative Research Institute for Innovative Microbiology, The University of Tokyo, Tokyo, Japan; Montana State University

## Abstract

This study reports the complete genome sequence of *Effusibacillus* sp. strain skT53. The genome is 3,454,394 bp in length and has a G+C content of 48.22 mol%.

## ANNOUNCEMENT

The genus *Effusibacillus* comprises three species, namely, Effusibacillus lacus (1), Effusibacillus pohliae (2), and Effusibacillus consociatus (3). The sources of these species are diverse, including geothermal soil on mountains, human blood, and freshwater lake sediments. We isolated *Effusibacillus* sp. strain skT53 from farm soil that supported poor crop growth in Adachi-ku, Tokyo, Japan (35°47′01.3″N, 139°47′58.0″E). Since it is the first strain from the genus *Effusibacillus* to be isolated from human-impacted soil, its genomic information can improve our understanding of the diversity of this genus.

Soil samples were collected from a depth of 10 cm and suspended in sterile saline solution. The supernatant was serially diluted and plated on SKT medium plates containing 0.8 mg Difco nutrient broth, 6 g gellan gum, and 0.16 g CaCl_2_ per liter. The inoculated plates were incubated at 37°C for 4 weeks. Nine microorganisms that showed growth on the SKT medium plate but not on the LB medium plate were selected. After confirmation of their ability to form spores, they were stored at −80°C in 15% (wt/vol) glycerol until further analysis. Of these nine strains, we selected the microorganism with the lowest level of similarity in the 16S rRNA gene sequence to strains with known sequences; we designated the strain skT53. The 16S rRNA gene sequence analysis indicated that E. consociatus CCUG53762^T^ (94.3%) (3), E. lacus skLN1^T^ (93.4%) (1), and E. pohliae MP4 (93.5%) (2) were most closely related to the skT53 strain.

The skT53 strain was cultured in yeast extract mineral liquid medium (DSM medium 259) (https://www.dsmz.de/collection/catalogue/microorganisms/culture-technology/list-of-media-for-microorganisms) at 50°C for 24 h. Genomic DNA from this strain was prepared from liquid medium-cultured cells using the Wizard genomic DNA purification kit (Promega, Madison, WI). The genome sequence was determined by Macrogen Japan Co., Ltd. (Kyoto, Japan) using a PacBio RS II system. The PacBio library was constructed using a SMRTbell template preparation kit (Pacific Biosciences, Menlo Park, CA). For size selection, the BluePippin size selection system (Sage Science, Beverly, MA) was used. Conditions for filtering the PacBio reads were as follows: minimum polymerase quality, 75; minimum subread length, 50 bases; and minimum polymerase read length, 50 bases. The statistics for the filtered reads were as follows: mean subread length, 9,597 bases; total number of subreads, 141,865; *N*_50_, 14,408 bases; and total subread size, 1,362 Mb. The read data were assembled using the FALCON-integrate assembler v. 2.14. Default parameters were used for all software. Assembly integrity was assessed with the Benchmarking Universal Single-Copy Orthologs (BUSCO) v. 3.0 software (https://busco.ezlab.org) (4) using the bacterial database odb9 and scored; the integrity was 98.7% (of a complete set of 148 orthologs). Assembly statistics were as follows: number of contigs, 1, and *N*_50_, 3,454,394 bases. It was confirmed by overlap that the genome is circular.

Genome sequence annotation was performed using the DDBJ Fast Annotation and Submission Tool (DFAST) (https://dfast.nig.ac.jp) (5); it showed 3,599 DNA coding sequences (CDSs), 27 rRNAs, 86 tRNAs, and one transfer messenger RNA.

### Data availability.

The GenBank/EMBL/DDBJ accession number for the genome sequence of strain skT53 is AP023366. The BioProject accession number is PRJDB10063, and the BioSample accession number is SAMD00233082. The raw sequence is available under DDBJ Sequence Read Archive (DRA)/NCBI SRA accession number DRR301952.
